# Does spontaneous renal hemorrhage mandate close surveillance for impending renal cell carcinoma? A case report and literature review

**DOI:** 10.1016/j.ijscr.2020.06.067

**Published:** 2020-06-20

**Authors:** Abdullah W. Aldughiman, Abdulrahman Alsunbul, Abdullah Al-Gadheeb, Muaiqel Almuaiqel, Ahmad Alzahrani, Tarek Alzahrani, Abdullah Alghamdi, Hamad Alakrash

**Affiliations:** Prince Sultan Military Medical City, Riyadh, Saudi Arabia

**Keywords:** Wunderlich syndrome, Renal cell carcinoma, Nephrectomy, Angioembolization, Computed tomography

## Abstract

**Introduction:**

Renal cell carcinoma (RCC) classically presents as a triad of hematuria, loin pain, and a palpable mass. However, Renal cell carcinomas (RCCs) nowadays are more commonly present as incidental findings rather than symptomatic. Wunderlich syndrome is a rare first presentation of RCC.

**Presentation of case:**

We present a clinical case of spontaneous renal hemorrhage with unclear etiology that was treated with therapeutic embolization and was found to have renal mass after long follow up.

**Discussion and conclusion:**

In regards to treating Wunderlich syndrome, some authors favor angioembolization and follow up. Others proposed radical nephrectomy in conditions with no apparent etiology and normal contralateral kidney because of the high incidence of small renal tumors. Spontaneous perinephric hematoma of unknown etiology should be followed up regularly with a CT image for concerning of impending renal tumor

## Highlights

• The spontaneous rupture of the kidney, however, is rarely the first clinical presentation of RCC.
• Neoplastic causes for Wunderlich syndrome accounted for 61.2% of these cases, with benign and malignant causes approximately divided equally.
• In treating Wunderlich syndrome, some urologists favor an early or immediate exploratory surgery, others prefer interventional radiology.
• Spontaneous perinephric hematoma of unknown etiology should be followed up regularly with CT image for concerning of impending renal tumor.

## 1 Introduction

Renal cell carcinoma (RCC) presents as asymptomatic incidental findings of renal mass [1]. The spontaneous rupture of the kidneys, or Wunderlich syndrome, is extremely rare phenomenon, but often the first manifestation of RCC. Herein, we present a clinical case of a patient with spontaneous renal hemorrhage due to unclear etiology, who was treated with therapeutic embolization; subsequently, a renal mass was identified after a long follow-up. The work reported herein adheres to the SCARE criteria [2].

## 2 Presentation of case

**Fig. 1:**
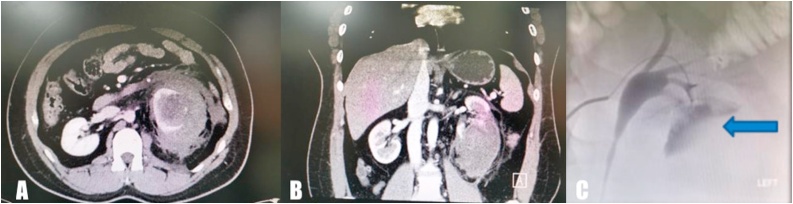
**A, B**: a computed tomography (CT) scan which revealed a 9.7 × 9 × 11 cm left perirenal hematoma mainly occupying the lower pole of the left kidney. **C:** angiogram during emergency embolization which revealed an area of extravasation.

A 39-year-old man presented to the emergency department with severe left flank pain. He reported a six-hour history of severe, non-radiating but constant pain, with a sudden onset, which progressively worsened. There was no history of trauma or stone disease. He did not have any previous history of medical disorder, and presently, was not on any medication. He was a heavy smoker (2 packs per day). On examination, his vital signs were stable. Abdominal examination showed generalized abdominal guarding and tenderness over the left costovertebral angle. Bowel sounds were present, and a poorly defined, palpable mass was present on the left side of the abdomen. Results of blood tests showed a decrease in serum hemoglobin levels (from 14.8 g/dL to 10.1 g/dL) within an hour, with a concomitant decrease in hematocrit level (from 37% to 29%). Intravenous fluid administration was initiated and two units of cross-matched blood was transfused. Thereafter, a computed tomography (CT) scan was performed, which revealed a 9.7 × 9 × 11 cm left perirenal hematoma mainly occupying the lower pole of the left kidney (Fig. 1A, B). The patient’s hemoglobin levels continued to decrease, for which, he was transfused with two additional units of blood. Serum creatinine level was normal. The patient was transferred to the interventional radiology suite, where he underwent an angiogram and embolization (Fig. 1C). He was admitted for close observation under strict bed rest. Moreover, a broad-spectrum antibiotic therapy was commenced.

After four days of observation and attaining a stable hemoglobin level, the patient was discharged home and scheduled for a follow-up abdomen CT scan in the outpatient clinic.

**Fig. 2:**
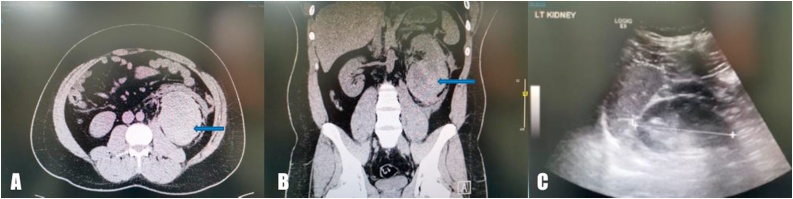
**A, B:** Follow up CT scan showed reduction of the hematoma by 50 percent compared to the initial CT scan, and there were no other abnormalities or lesions. **C:** Follow up renal Ultra sound showed no masses, stones or hydronephrosis with normal corticomedullary differentiation.

The follow-up CT scan showed reduction of the hematoma by 50 percent as compared with the initial CT scan, and no other abnormality or lesion was noted (Fig. 2A, B). The patient was completely asymptomatic, with stable hemoglobin levels on follow-up. Three months after discharge, the patient underwent a kidney ultrasound, which showed no mass, stone, or hydronephrosis; normal corticomedullary differentiation was observed (Fig. 2C).

**Fig. 3:**
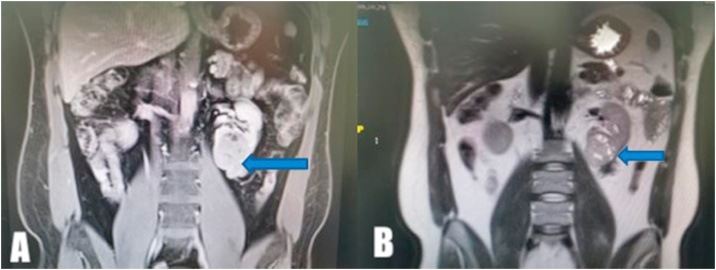
MRI, which showed left renal lower pole heterogenous mass measuring 6 × 5 × 4 cm suspicious for renal cell carcinoma.

The patient was routinely followed up with serial ultrasounds for two years, and the cause of the perirenal hematoma could not be identified. Therefore, a decision was made to perform an abdominal MRI, which showed a heterogenous mass measuring 6 × 5 × 4 cm in the left renal lower pole; the mass was suspected to be a RCC (Fig. 3A, B)

**Fig. 4:**
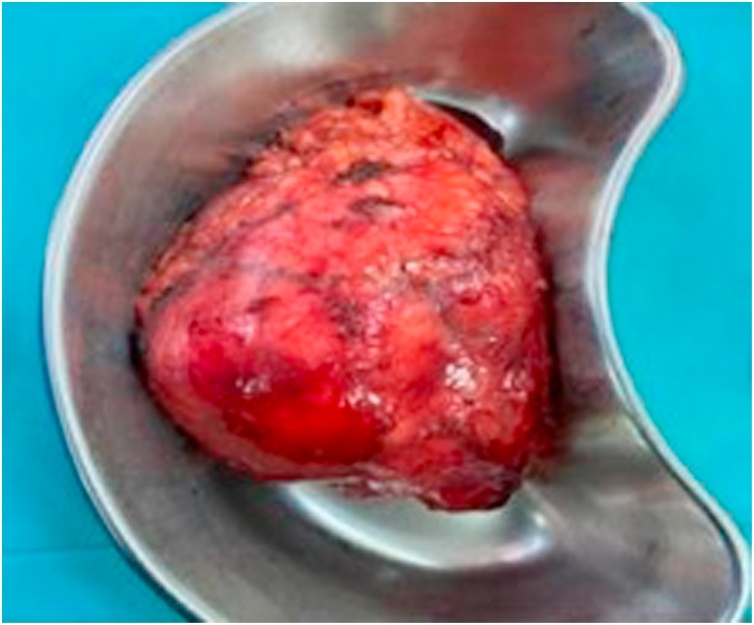
Gross specimen of the left kidney contained within the Gerota Fascia.

Thus, a left laparoscopic radical nephrectomy was performed with no perioperative complications (Fig. 4). The patient had an uneventful recovery and was discharged home three days postoperatively.

Results of histological examination of the surgical specimen revealed chromophobe RCC, which was 5.8 cm in its largest dimension, negative for sarcomatoid and rhabdoid features, negative for tumor necrosis, but positive for lymphovascular invasion. The tumor was assigned a pathological stage of pT3a pNx cM0.

## 3 Discussion

With the advent in sophisticated imaging techniques, the classical presentation of loin pain, palpable mass, and frank hematuria are less commonly seen in clinical settings. However, RCCs are now more commonly discovered as ‘incidentalomas’ on radiological imaging for alternative abdominal pathologies [1]. The spontaneous rupture of the kidneys, however, is rarely the first clinical presentation of RCC. In 1856, Carl Reinhold August Wunderlich described spontaneous bleeding of the kidney with dissection of blood into the subcapsular and/or perinephric spaces [3]. Wunderlich syndrome is rare and usually caused by a benign disease [4]. The most recent meta-analysis by Zhang et al., in 2002, reviewed the cases of 165 patients with spontaneous perirenal hemorrhage due to various causes, reported between 1985 and 1999 [5]. In 70% of these patients, the hemorrhage occurred due to benign causes, including vascular disease, infection, and neoplasia. Overall, neoplastic etiologies accounted for 61.2% of these cases, and benign and malignant causes had approximately equal contribution.

In the treatment of Wunderlich syndrome, some urologists favor an early or immediate exploratory surgery [6]. Other urologists, however, prefer interventional radiology-based approaches to stop an acutely hemorrhaging vessel [7]. There are logical reasons to avoid surgery in acute cases, such as when the patient’s general condition is not fully stabilized; however, embolization with the intention of delayed surgery further complicates surgical resection. Currently, there are no guidelines favoring either approach.

Another concern is the efficiency of CT to diagnose renal tumors at the time of bleeding. CT remains one of the most reliable modalities to diagnose retroperitoneal hemorrhage and RCCs [8]. Kendall et al., however, reported that 60% of the patients with renal hemorrhage had an undiagnosed RCC at the time of initial CT scan [9]. This observation was similar to our present case and was in agreement with the observations of Zhang et al.’s meta-analysis, which reported that CT performed at the time of hemorrhage was only partially efficient at identifying renal tumors (sensitivity, 57%) [5]. There are two approaches to manage such cases. In the cases with no apparent etiology and a normal contralateral kidney, Kendall et al. proposed radical nephrectomy as a treatment of choice, with careful pathological examination of the surgical specimens because of the high incidence of small renal tumors [9].

In contrast, Morgentaler et al. proposed nephrectomy only in patients with nonfatty lesions other than hematoma. All other patients were followed up with serial CT scans [9]. Bosniak et al. claimed that operative exploration is not necessary in most unexplained cases of renal hemorrhage because of the superior diagnostic accuracy of contrast-enhanced CT scans with 5-mm sections [5]. If the etiology cannot be determined at primary examination, a follow-up CT scan should be performed with at least 3-month intervals until the hematoma resolves and a definite diagnosis is possible.

For malignant tumors diagnosed on initial CT, radical nephrectomy is eventually required. However, in the renal hemorrhages thought to be benign, embolization may be the sole modality used. If these patients were later found to have a malignancy on the follow-up CT, delayed surgery would not only affect the overall resect ability of the tumor, but also the clinical staging of the disease [10,11].

When CT is contraindicated (e.g., in patients with contrast agent sensitivity or renal insufficiency), magnetic resonance imaging (MRI) may be useful, especially for follow-up after an initial improvement in acute hemorrhage. Even if a patient undergoes multi-modal imaging both before and after evacuation of the hematoma, the tumor causing the hemorrhage may be extremely small to be visualized by CT or angiography. This finding was observed in one patient in our series in whom the initial imaging (performed both before and after the evacuation of the hematoma) did not show any abnormality. The patient presented 1 year later with hematuria, and follow-up imaging demonstrated a small, lower pole RCC, which was similar to our present case [10,11]. Previously, some authors have recommended that a radical nephrectomy should be performed in all patients with spontaneous renal hemorrhage in whom nonneoplastic causes have been excluded [9]. Presently, however, multiphasic helical CT scans and rapid MRI sequences have greatly reduced the need for exploratory surgery in the diagnosis, follow-up, and treatment of patients with spontaneous renal hemorrhage.

Moreover, the pathological type of the RCC plays a major role in tumor growth and progression. In our case, one of the explanations that the chromophobe tumors had slow progression rate compared to other types.

## 4 Conclusion

Patients with spontaneous perinephric hematoma due to unknown etiology should be followed up regularly with CT scans to assess the risk of an impending renal tumor. If the etiology cannot be ascertained at initial examination, a follow-up CT scan should be performed with at least 3-month intervals until the hematoma resolves and a definite diagnosis is possible. Furthermore, MRI might be useful to diagnose the uncertain findings in such patients.

## Declaration of Competing Interest

No conflict of interest.

## Funding

This research did not receive any specific grant from funding agencies in the public.

## Ethical approval

Case reports are exempted from IRB/ethical approval at the institution.

## Consent

Patient has given his consent for the case report to be published. A copy of the written consent is available, at anytime.

## Author contribution

Abdullah W. Aldughiman: draft of the manuscript and primary author.

Abdulrahman Alsunbul: draft of the manuscript and review of the literature.

Abdullah Al-Gadheeb: grammar and spelling and review of the literature.

Muaiqel Almuaiqel: collection of figures.

Ahmad Alzahrani: review of the literature.

Tarek Alzahrani: review of the literature.

Abdullah Alghamdi: review of the literature.

Hamad Alakrash: primary consultant & Surgeon final modification.

## Registration of research studies

NA.

## Guarantor

NA.

## Provenance and peer review

Not commissioned, externally peer-reviewed.
